# Dual-branch hierarchical fusion network for voice-based classification of type 2 diabetes mellitus using multi-vowel acoustic features

**DOI:** 10.3389/fmedt.2026.1908856

**Published:** 2026-09-03

**Authors:** Pengxu Jiang, Wenke Zhang, Aiqin Li, Peng Li

**Affiliations:** 1 Central Hospital of Kaifeng, Kaifeng, China; 2 The Institute of Complexity Science, Henan University of Technology, Zhengzhou, China

**Keywords:** convolutional neural networks, deep neural networks, non-invasive T2DM, speech features, T2DM detection

## Abstract

**Problem:**

While voice-based analysis has emerged as a potential non-invasive approach for exploring acoustic alterations associated with Type 2 Diabetes Mellitus (T2DM), current approaches often fail to fully capture disease-relevant vocal patterns due to simplified recording protocols and insufficient feature modeling.

**Aim:**

This study aims to investigate the feasibility of voice-based classification of T2DM using a multi-feature fusion framework, leveraging sustained vowel phonations and integrating multiple acoustic feature types to enhance diagnostic performance.

**Methods:**

Voice recordings were collected from 378 participants, including T2DM patients and healthy controls. Each participant pronounced six Mandarin vowels, from which acoustic features—including Mel-frequency cepstral coefficients (MFCCs), glottal parameters, and eGeMAPS descriptors—were extracted. Neural networks, including convolutional and deep architectures, were applied to capture pathology-relevant segments. Vowel-level fusion modules aggregated features across vowels, and an attention-based hierarchical fusion mechanism combined the three feature types, adaptively emphasizing features most relevant to T2DM.

**Results:**

The proposed dual-branch hierarchical fusion model achieved 80.48% detection accuracy.

**Conclusion:**

These results suggest that sustained vowel phonations contain potentially informative acoustic patterns associated with T2DM status. However, further validation using independent cohorts is required before clinical application.

## 1 Introduction

Type 2 Diabetes Mellitus (T2DM) is a chronic metabolic disease marked by insulin resistance and hyperglycemia, often progressing asymptomatically in its early stages (1). Traditional diagnostic approaches typically rely on biochemical blood tests such as fasting plasma glucose or HbA1c measurements, which are time-consuming, inconvenient, and difficult to scale for population-level screening (2). In recent years, non-contact T2DM detection methods have gained increasing attention due to their potential for cost-effective, rapid, and user-friendly screening (3). Among them, voice-based T2DM detection (4) has emerged as a novel paradigm that leverages the physiological link between metabolic conditions and vocal production mechanisms. Specifically, T2DM can affect neuromuscular control, vocal fold tension, and respiratory capacity, leading to subtle alterations in speech signals. This suggests that speech signals may contain acoustic characteristics associated with T2DM-related physiological changes. Such approaches may provide a foundation for future non-invasive screening studies after further validation.

Despite the potential, existing voice-based T2DM studies often face several challenges. First, most existing works have focused exclusively on single vowels and have overlooked the acoustic variations introduced by different phonation patterns (5, 6). Additionally, current efforts in voice-based T2DM detection tend to rely on identifying statistically significant low-dimensional features (5, 7). which are often insufficient to capture the complex pathological characteristics of T2DM. Given the demonstrated effectiveness of relevant acoustic pathological features, it is therefore necessary to consider a comprehensive combination of multiple feature types (8). Furthermore, existing work rarely translates these discriminative features into systematic and automated diagnostic frameworks, thereby significantly limiting their potential for clinical deployment and practical application in diabetes screening. Given the widespread application of neural networks in non-invasive diabetes detection (9), it is essential to develop specialized network architectures tailored for voice-based T2DM detection.

**Figure 1:**
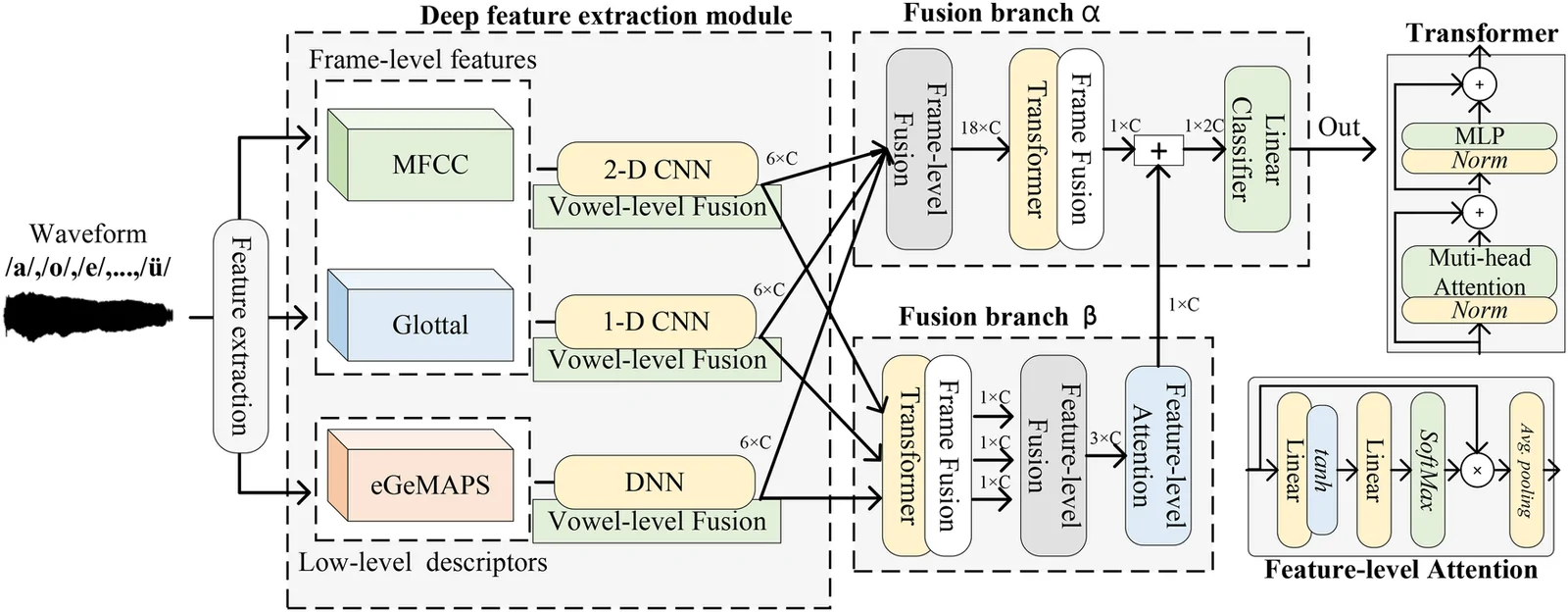
Illustration of the proposed architecture for voice-based T2DM detection.

In this study, we propose a multi-feature fusion strategy for voice-based T2DM detection. As illustrated in Figure 1, our protocol involves collecting six Mandarin vowel phonations (/a/, /e/, /i/, /o/, /u/, /ü/) from each participant to ensure phonetic coverage. This strategy overcomes the limitation of previous studies by incorporating multiple vowel phonations, thereby capturing the acoustic differences across vowels and enabling a more comprehensive representation of disease-related speech patterns. Subsequently, we extract three complementary acoustic feature sets, comprising two frame-level features and low-level descriptors. For frame-level acoustic analysis, we extracted both Mel-frequency cepstral coefficients (MFCCs) (10) and glottal features (Glottal) (11). MFCCs were employed to capture the time-frequency characteristics of vowel sounds. The glottal features reflect phonatory characteristics, particularly glottal closure efficiency, vocal fold tension, and airflow control. Subsequently, we extracted eGeMAPS features (12), a standardized set commonly used in automatic speech analysis and clinical voice research. This multi-feature extraction approach combines complementary high-dimensional acoustic features, enabling a more comprehensive representation of the complex pathological voice characteristics associated with T2DM.

In order to achieve automated prediction of T2DM, we developed a hierarchical deep architecture consisting of a dedicated deep feature extraction module and a dual-branch fusion branch. Specifically, 1D-CNNs are employed to model temporal dependencies within specific dimensions of Glottal waveform features. The 2D-CNNs are designed to extract localized time–frequency representations from MFCCs, effectively leveraging the structured spectral patterns associated with vocal tract changes. Meanwhile, the DNNs are utilized to learn high-level global abstractions from the eGeMAPS features, enabling the integration of prosodic, spectral, and voice quality descriptors relevant to T2DM-related vocal alterations. The three fused feature representations are subsequently fed into two complementary fusion branches.

In fusion branch α, the three feature representations are first combined through a frame-level fusion layer, followed by a Transformer encoder to capture vowel-level dependencies across different feature types. In fusion branch β, each feature representation is initially processed independently by a Transformer encoder, after which a feature-level fusion layer is applied to integrate cross-vowel feature information, and a feature-level attention layer is subsequently employed to fuse heterogeneous feature types. The output of fusion branch β is finally integrated with that of fusion branch α to generate the final prediction for binary classification. This neural network–based framework enables automated voice-based T2DM detection by integrating diverse acoustic features into a unified predictive model.

The experimental phase demonstrates the feasibility of the proposed architecture for voice-based T2DM detection and establishes a foundation for non-invasive, voice-based diagnostic systems. By integrating temporal, spectral, and statistical vocal cues, this method advances the application of speech analysis in chronic disease screening.

The main contributions of this paper can be summarized as:

- We designed a multi-vowel voice recording protocol for T2DM detection, to comprehensively represent disease-related acoustic variations.
- We proposed a dual-branch hierarchical fusion model for experimental classification of T2DM based on multi-vowel acoustic representations.
- The proposed model achieved an overall detection accuracy of 80.48%, demonstrating the feasibility of multi-feature fusion for voice-based T2DM classification within the investigated cohort.

The remainder of this paper is organized as follows. Section 2 describes the proposed speech recording protocol and feature extraction pipeline, Section 3 presents the design of the proposed model, and Section 4 details the experimental setup and the analysis of the results.

## 2 Methodology

### 2.1 Data collection

To investigate whether T2DM affects vocal production, we recruited a total of 378 participants from Kaifeng Central Hospital, including 192 individuals diagnosed with T2DM and 186 healthy controls. The cohort consisted of 190 females and 188 males. It should be noted that each participant contributed six independent speech recordings, resulting in a total of 2,268 utterances. The participant was defined as the fundamental unit of analysis. Six vowel recordings from each participant were first processed independently and subsequently fused to generate one final prediction per participant. This study received approval from the ethics committee of kaifeng central hospital (number: 2024ks-kt024), and all participants provided signed informed consent prior to enrollment. All procedures were conducted in accordance with relevant guidelines and regulations. To ensure participant protection, all speech recordings and associated data were anonymized. Specifically, fasting plasma glucose (FPG) or glycated hemoglobin (HbA1c) levels were used to distinguish the ND group from the T2DM group. T2DM was defined based on clinical diagnosis and laboratory measurements, including FPG≥7.0 mmol/L or HbA1c≥6.5%. The control group consisted of individuals without diabetes according to medical records and laboratory examination. Participants with known respiratory diseases, neurological disorders, severe cardiovascular diseases, or any acute conditions that could affect speech production were excluded from the study.

Before recording, the researchers provided pronunciation guidance to all participants to ensure correct pronunciation of six standard Mandarin vowels (/a/, /o/, /e/, /i/, /u/, and /ü/). Speech recordings were collected using a dual-channel MIC6 microphone with a sampling rate of 16 kHz, positioned approximately 10–15 cm from the speaker’s lips at a 45-degree angle. All recordings were conducted in a relatively quiet, real-world hospital environment. Each vowel was recorded three times (3–4 s per recording). Instead of manually selecting the most coherent recording, recordings were evaluated according to predefined quality-control criteria, including signal-to-noise ratio, recording duration, clipping detection, and pronunciation completeness. Immediately following the recordings, preliminary auditory screening was conducted by research staff to exclude low-quality samples. The remaining recordings were saved in WAV format. To ensure data security, all audio files were encrypted and uploaded to a secure server, accessible only to authorized research personnel via a private key authentication system.

### 2.2 Feature extraction

To effectively characterize the acoustic manifestations associated with T2DM, we designed a multi-perspective feature extraction framework comprising three complementary categories of speech features: acoustic statistical functionals, glottal source features, and spectrum features. Specifically, acoustic statistical functionals provide a standardized and compact representation of vocal attributes. Frame-level glottal features are employed to detect laryngeal dysfunctions by modeling vocal fold behavior and subglottal pressure changes, while spectrum features capture the time–frequency patterns of vowel articulations. The combination of these three categories aims to offer a holistic and physiologically interpretable representation of voice signals, facilitating accurate and robust T2DM prediction. The detailed descriptions of each feature group are provided as follows:

**eGeMAPs features**: To characterize global voice quality and paralinguistic attributes, we utilized the extended Geneva Minimalistic Acoustic Parameter Set (eGeMAPS), extracted via the openSMILE toolkit (13). Specifically, the eGeMAPS features consist of statistical functionals computed from a set of low-level acoustic descriptors (LLDs) extracted at the frame-level. They comprises 88 low-level descriptors and statistical functionals, including several parameters such as pitch, jitter, and shimmer that have been previously demonstrated to be significantly associated with T2DM in earlier works. In addition, eGeMAPS encompasses energy-related features (e.g., loudness), spectrum characteristics (e.g., spectral slope), and other relevant acoustic descriptors, as shown in Table 1. In our study, eGeMAPS summarizes the overall voice characteristics across each vowel utterance, offering indicators of vocal fatigue or instability possibly linked to diabetic symptoms.

**Table 1:** eGeMAPs feature categories.

Category	eGeMAPS features
Energy/amplitude	loudness; sound Level; HNR; shimmer
Frequency	F0; Jitter; Formants 1–3 frequency and bandwidth
Spectral balance	Alpha ratio; Hammarberg index; Spectral slope;
	MFCCs 1-4; H1-H2 and H1-A3; Spectral flux
Temporal	Rate of loudness peaks; Mean length and SD of Voiced and unvoiced segments; syllable rate

**Glottal features**: To investigate subtle laryngeal alterations associated with T2DM, we extracted a set of frame-level glottal features that directly reflect vocal fold dynamics. The audio signals were divided into frames with a 200-ms frame length and a 100-ms frame shift for feature extraction. These features were derived from glottal inverse filtering techniques and include 12 parameters, as shown in Table 2. These parameters capture both the temporal and spectral characteristics of glottal excitation. For each glottal feature, we computed the mean and standard deviation of these parameters, resulting in a 24-dimensional feature vector per frame. Furthermore, to enhance temporal contextual representation, we calculated the first-order and second-order derivatives of the glottal parameters, ultimately obtaining 72-dimensional glottal features for each frame.

**Table 2:** Glottal parameters used as features in the experiments.

Time-domain glottal parameters	
GCId	glottal closure instants duration,
NAQ	normalized amplitude quotient,
QOQ	quasi open quotient
OQ1	open quotient, calculated from the primary glottal opening
OQ2	open quotient, calculated from the secondary glottal opening
SQ1	Speed quotient, caleulated from the primary glottal opening
SQ1	Speed quotient, caleulated from the secondary glottal opening
AQ	amplitude quotient
CIQ	closed phase quotient
Frequency-domain glottal parameters	
HRF	harmonic richness factor
H1H2	difference between the first two harmonics of the glottal flow signal
PSP	parabolic spectral parameter

**MFCCs**: MFCCs are used to capture the time-frequency patterns of vowels. Due to their effectiveness in representing spectral envelope variations, MFCCs have become a standard tool in speech signal processing and medical diagnostics. MFCCs were computed for each 200-ms speech frame using a 100-ms shift. Specifically, the first 36 coefficients were extracted as feature parameters for each frame.

## 3 Neural networks for feature modeling

In this study, we propose a parallel architecture designed to process multiple speech features independently and simultaneously. Specifically, the proposed framework consists of a Deep Feature Extraction Module and two fusion branches. Each model is described in detail below, outlining its unique capabilities and contribution to the overall prediction of T2DM.

### 3.1 Deep feature extraction module

The Deep Feature Extraction Module primarily consists of a 2-D CNN, 1-D CNN, and a DNN. DNNs are employed to process the eGeMAPS feature set, which provides a global representation of voice quality and prosody. Specifically, the DNN comprises three fully connected layers with 32, 64, and 128 neurons, respectively.

To effectively capture local structures in sequential acoustic features, we introduce two types of Convolutional Neural Networks (CNNs) in our parallel architecture. The 1D-CNN is applied to the glottal frame-level feature set, which preserves the temporal structure of speech. The 1D-CNN model applies one-dimensional convolutional kernels along the time axis to extract short-term temporal dependencies, such as micro-variations in glottal flow that may be influenced by neuromuscular changes. The 2D-CNN is employed to process the MFCC features. Unlike the sequential nature of the glottal frame-level feature set, MFCCs provide a spectro-temporal representation of speech. Convolutional layers in a 2D CNN learn spatial features across both time and frequency, allowing the network to capture complex patterns that may indicate underlying changes in vocal tract control associated with T2DM. The architectures of the proposed 1D-CNN and 2D-CNN models are summarized in Tables 3, 4. a global average pooling (GAP) layer and a dense layer are used for feature mapping. Batch normalization and ReLU activation are applied following every convolutional layer. Additionally, all models are followed by a Vowel-level Fusion layer, which is used to perform frame-level aggregation of features across multiple vowels. Specifically, for the six vowel phonations, the output dimension of all modules is 6×C, where C=128.

**Table 3:** Proposed 1D-CNN.

Layer	Description	Output size
Input	/	T×72×1
Conv	32, 5×1, stride:2×1	T/2×72×32
Conv	32, 5×1, stride:1×1	T/2×72×32
AVG pooling	2×1, stride:2×1	T/4×72×32
Conv	64, 5×1, stride:1×1	T/4×72×64
Conv	64, 5×1, stride:1×1	T/4×72×64
AVG pooling	2×1, stride:2×1	T/8×72×64
Conv	128, 3×1, stride:1×1	T/8×72×128
Conv	128, 3×1, stride:1×1	T/8×72×128
AVG pooling	(T/8)×1, stride:(T/8)×1	72×128
Dense	128	

**Table 4:** Proposed 2D-CNN.

Layer	Description	Output size
Input	/	T×F×1
Conv	32, 5×5, stride:2×2	T/2×F/2×32
Conv	32, 5×5, stride:1×1	T/2×F/2×32
AVG pooling	2×2, stride:2×2	T/4×F/4×32
Conv	64, 3×3, stride:1×1	T/4×F/4×64
Conv	64, 3×3, stride:1×1	T/4×F/4×64
AVG pooling	2×2, stride:2×2	T/8×F/8×64
Conv	128, 3×3, stride:1×1	T/8×F/8×128
Conv	128, 3×3, stride:1×1	T/8×F/8×128
GAP	128	

### 3.2 Fusion branches

These three feature representations are subsequently fed into two complementary fusion branches, namely fusion branch α and fusion branch β, which are designed to model cross-feature interactions and vowel-level dependencies from different perspectives.

In fusion branch α, the three feature representations are first combined through a frame-level fusion layer. This layer performs feature concatenation across the frame dimension, enabling the model to integrate heterogeneous acoustic cues into a unified representation. Specifically, the two linear layers within the Feature-level Attention module do not alter the shape of the input features. The fused representation is then passed to a Transformer encoder, which is employed to capture long-range contextual dependencies and interactions among different feature types across vowel frames. By leveraging the self-attention mechanism, the Transformer enables the model to model global relationships within the fused feature sequence and to learn discriminative patterns relevant to T2DM-related vocal variations. The Transformer architecture is primarily composed of multi-head self-attention, the mathematical formulation of this mechanism can be represented as (Equations 1, 2):

$$h_{i}=\mathrm{Attention}(Q,K,V)=\mathrm{softmax}\left(\frac{QK^{T}}{\sqrt{d_{k}}}\right)V$$ (1)

$$\mathrm{MultiHead}(Q,K,V)=\mathrm{Concat}\left(h_{1},h_{2},\ldots ,h_{h}\right)W^{O}$$ (2)

where Q, K, V denote the query, key, and value matrices respectively, and $d_{k}$ is the dimension of the key vector. Subsequently, the frame fusion layer applies frame-level average pooling to obtain the output representation of the fusion branch α.

In Fusion Branch β, the three feature representations are first processed independently by separate Transformer encoders. This design allows each feature modality to learn its own contextual representation and temporal dependencies before cross-feature interaction occurs. After the independent encoding process, the resulting representations are each passed through a frame-level fusion layer to obtain separate feature vectors. Subsequently, a feature-level attention layer is applied to the fused representation. This layer assigns adaptive weights to different feature-type representations, enabling the model to emphasize features that contain stronger pathological cues associated with T2DM. Finally, the output of Fusion Branch β is fused with the output of Fusion Branch α, producing the final integrated representation of the proposed architecture. This representation is then fed into a classification layer to generate the final prediction for the binary T2DM detection task.

## 4 Results

### 4.1 Training setup

The input features for our model comprised the eGeMAPS feature set, glottal features, and Mel-frequency cepstral coefficients (MFCCs), with the same feature extraction scheme applied to six vowel categories. Frame-level features were extracted using a 16 kHz sampling rate, 200-ms frame length and 100-ms frame shift. The MFCCs consisted of 36 frequency dimensions, while the eGeMAPS features had a fixed dimension of (1×88). The glottal features and MFCCs were structured in dimensions of (N×72) and (N×36), respectively, where *N* denotes the number of frames.

During training, model parameters were optimized using the Adam optimizer with a batch size of 32 and 128 epochs, the learning rate is set to 0.0001. The Transformer encoder had 8 attention heads and 4 layers. To enhance training stability and reduce the risk of overfitting, L2 regularization and batch normalization were applied to all convolutional layers, and dropout (0.3) was applied following the fully connected layers. To ensure robust evaluation, we performed 5-fold cross-validation with independent speaker groups to assess the proposed framework. In each fold, the subjects used for training and testing were completely disjoint, ensuring that no speaker appeared in both sets. The proposed model was implemented using TensorFlow. The Transformer encoder consisted of 4layers with 4 attention heads. The embedding dimension was set to 128. All preprocessing procedures, including feature normalization and parameter estimation, were performed independently within each training fold.

### 4.2 Comparative analysis using eGeMAPS across multiple methods

To investigate the statistical association between acoustic features and T2DM, an independent-samples t-test was first conducted. Specifically, several features from the eGeMAPS feature set that have been reported to be significantly related to T2DM were examined, including F0, HNR, localJitter, and localShimmer. The detailed results are presented in Table 5. This analysis aims to examine whether these acoustic features exhibit significant differences between T2DM patients and healthy controls, thereby validating their feasibility as potential discriminative features for T2DM detection.

**Table 5:** Statistical comparison of acoustic features across different phonation types.

p	/a/	/o/	/e/	/i/	/u/	/ü/
F0	**<0.05**	0.47	**<0.05**	**<0.05**	**<0.05**	0.76
HNR	0.41	**<0.05**	0.18	0.30	**<0.05**	0.29
localJitter	**<0.05**	**<0.05**	**<0.05**	**<0.05**	0.52	**<0.05**
localShimmer	**<0.05**	0.27	0.87	0.63	**<0.01**	0.15

Bold values p < 0.05 indicates that the feature is statistically significant.

The statistical results presented in Table 5 indicate that several acoustic features exhibit significant differences between T2DM patients and healthy controls across different vowel phonations, these results suggest that several acoustic features derived from the eGeMAPS set demonstrate significant statistical associations with T2DM, supporting their effectiveness as potential discriminative features for T2DM detection.

Given that one-dimensional acoustic features can be processed through various approaches, we evaluated the performance of the eGeMAPS feature set when used as input to different models. The comparison included both conventional machine learning algorithms and the proposed DNN architecture. For the machine learning baseline, we selected k-nearest neighbors (KNN), support vector machines (SVM) and and logistic regression (LR), implemented via the scikit library in Python with default parameter settings to ensure reproducibility. Table 6 presents the experimental results, which include prediction outcomes across multiple vowel input features.

**Table 6:** Comparative evaluation of model performance (%) on the eGeMAPS feature set.

Method	Speech task					
	/a/	/o/	/e/	/i/	/u/	/ü/
SVM	59.12	62.60	62.93	60.05	60.35	61.00
KNN	54.61	57.70	55.87	53.32	57.82	55.22
LR	52.64	60.84	52.64	50.79	57.17	55.29
DNNs	70.04	68.46	69.12	67.56	67.92	70.23

The comparative evaluation of model performance on eGeMAPS-based vowel classification reveals two key findings. First, the proposed DNN architecture demonstrates statistically significant superiority over conventional machine learning approaches, achieving a high classification accuracy of 70.23% compared to 62.93% for SVM and 57.82% for KNN. This performance advantage is particularly pronounced for the vowels /a/ and /ü/, where the DNN achieves an average performance improvement of 10.07% compared to the SVM, demonstrating its superior capability in learning the complex structural relationships among these acoustic features. Second, the DNN consistently outperformed both the SVM and KNN across all six vowel categories. The largest absolute performance difference was observed for /a/ (DNN: 70.04% vs. KNN: 54.61%), while the smallest difference occurred for /o/ (DNN: 68.46% vs. SVM: 62.6%). These findings collectively demonstrate that deep learning architectures exhibit a clear advantage in processing one-dimensional acoustic features, particularly when dealing with acoustically complex feature sets.

#### 4.2.1 The influence of input feature parameters on 1D-CNN and 2D-CNN

**Figure 2:**
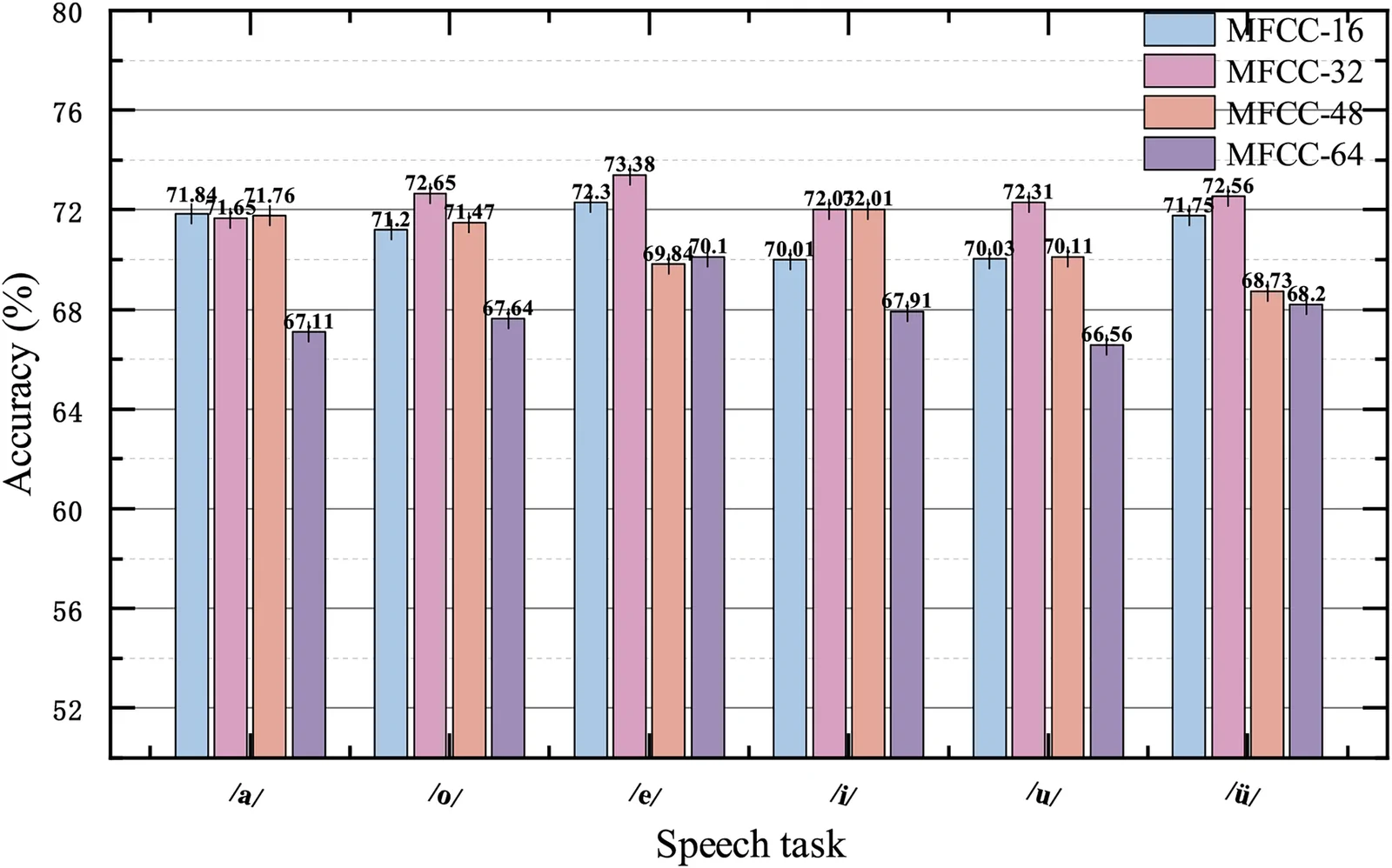
Performance testing of MFCC in 2D-CNN.

**Figure 3:**
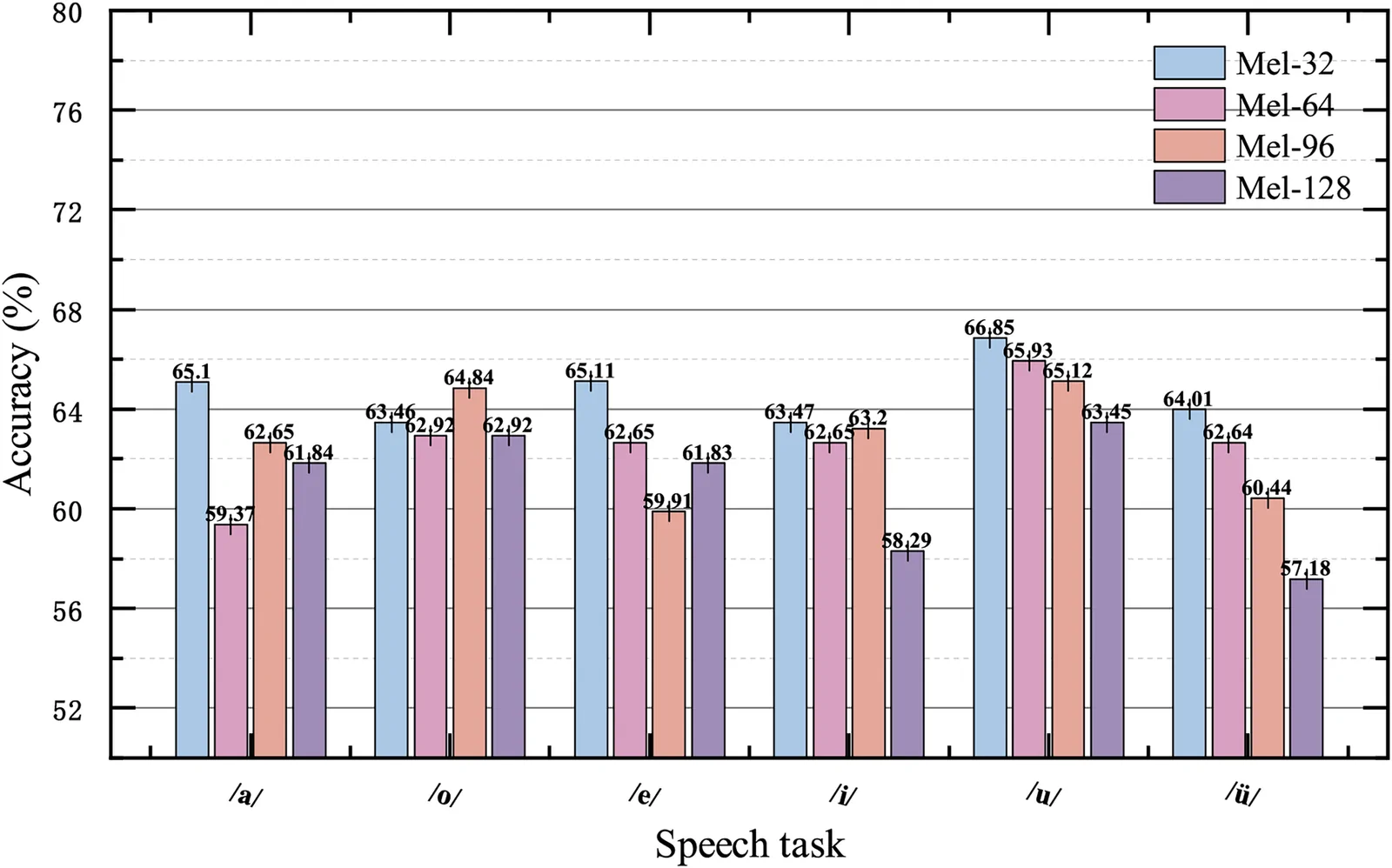
Performance testing of log-Mel in 2D-CNN.

**Figure 4:**
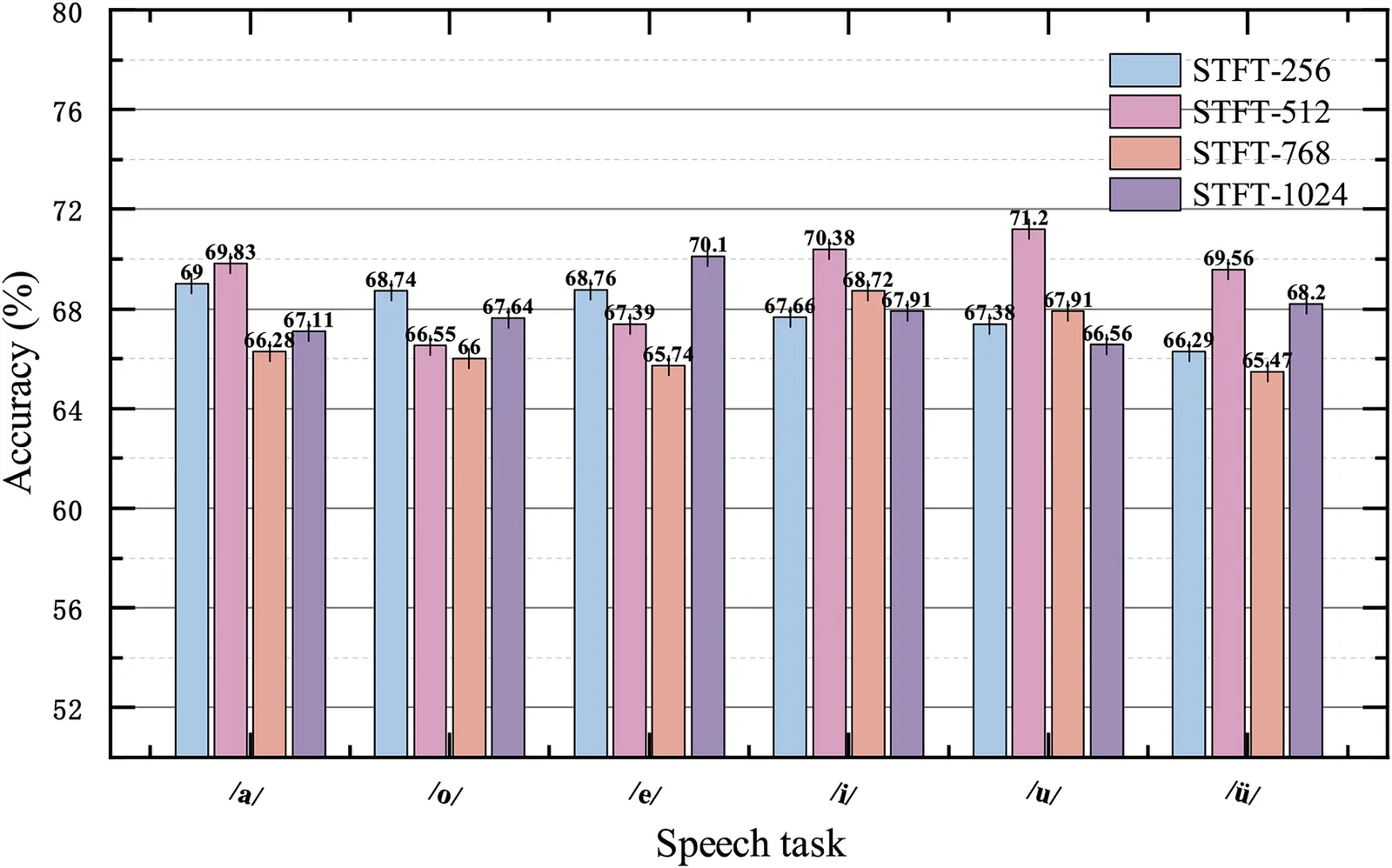
Performance testing of STFT in 2D-CNN.

We first evaluated the performance of various time–frequency features using 2D-CNN. Specifically, the features included MFCC, log-Mel spectrograms, and short-time Fourier transforms (STFT). To account for the influence of parameter configurations on model performance, we examined multiple frequency resolutions: for MFCCs, the number of bins ranged from 16 to 64; for log-Mel spectrograms, the number of Mel bins ranged from 32 to 128; and for STFTs, the window size varied from 256 to 1024. The experimental results are presented in Figures 2–4, along with a performance comparison across different vowel pronunciation tasks.

As summarized in Figures, the experimental results demonstrate that MFCC-based features consistently outperform log-Mel spectrograms and STFT representations across all vowel pronunciation tasks. For MFCC, the optimal performance was generally achieved with bin numbers in the range of 16–48, with the /e/ vowel reaching its highest accuracy of 73.38% at MFCC-32. Although performance exhibited slight fluctuations as the bin count increased, MFCC features maintained higher average accuracies compared to the other feature types. In contrast, log-Mel spectrogram features yielded lower overall accuracy, with peak performance observed at relatively low Mel bin counts (e.g., 66.85% for /u/ with 32 Mel bins). Increasing the number of Mel bins beyond this point did not produce performance gains. Similarly, STFT features exhibited moderate performance, with accuracies generally falling between those of MFCC and log-Mel. While certain vowels, such as /i/ and /u/, achieved competitive results with small window sizes, the overall average performance remained lower than that of MFCC-based features. This may be attributed to the excessively large frequency parameters, which hinder the CNN’s ability to fully learn the underlying patterns. These findings indicate that MFCCs provide more robust and discriminative time–frequency representations for vowel-based T2DM classification in a 2D-CNN framework. The superior performance of MFCCs may be attributed to their ability to compactly capture perceptually relevant spectral cues while suppressing redundant information, thereby facilitating more effective feature learning by convolutional architectures.

**Figure 5:**
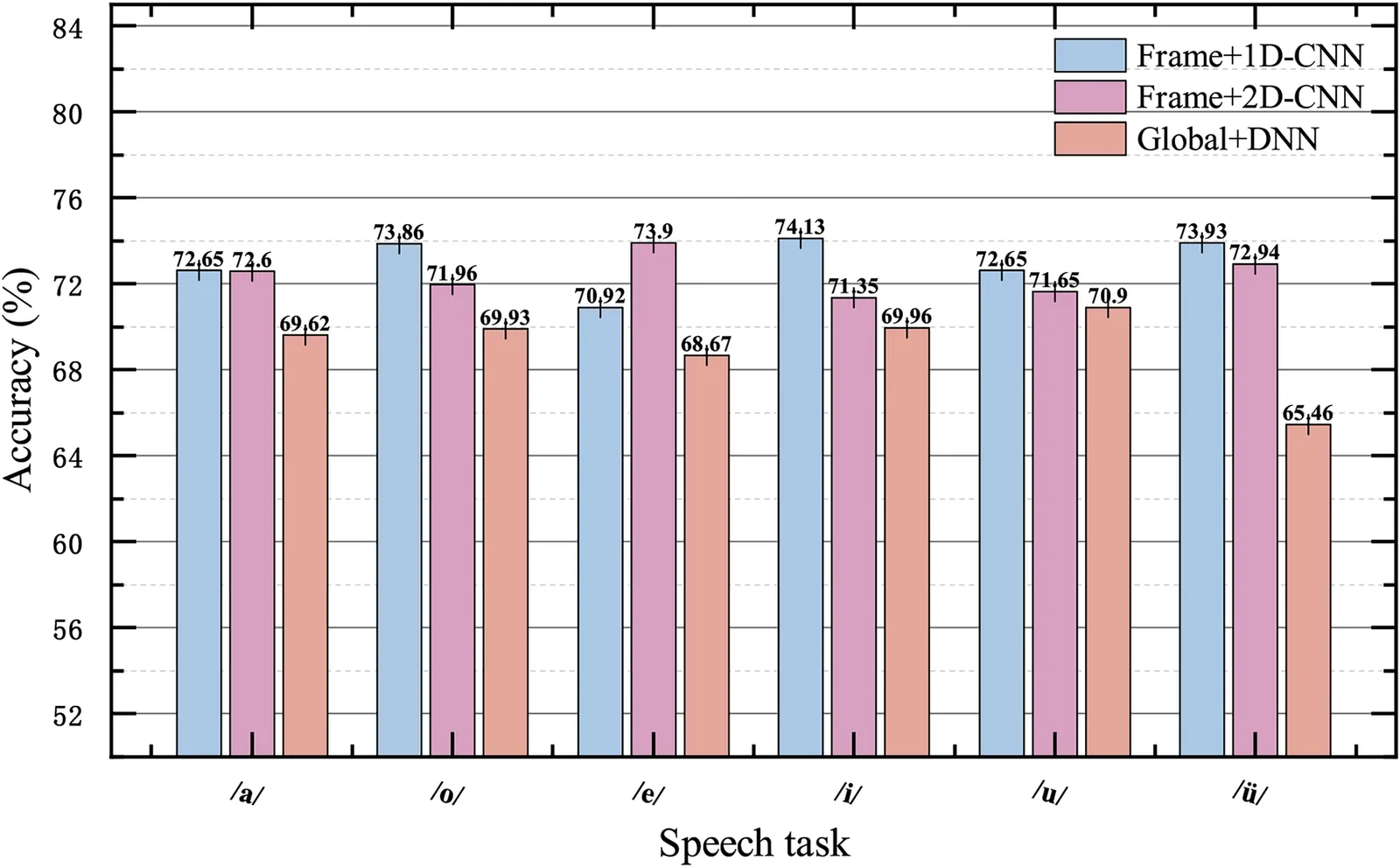
Performance testing of glottal in 1D-CNN.

Subsequently, we evaluated the performance of Glottal features using 1D-CNN. In addition, the 2D-CNN was employed as an alternative. To further examine the difference between local and global vowel Glottal features, we computed the global average of frame-level features and used these as inputs to the DNN. The experimental results, summarized in Figure 5, also provide a comparative analysis of performance across different vowel categories.

As shown in Figure, the frame-level glottal features processed by the 1D-CNN achieved superior performance compared to both the 2D-CNN and the DNN models. The 1D-CNN applies temporal convolution along the sequential frames and is well-suited for capturing temporal patterns inherent in the glottal features. In contrast, replacing the 1D-CNN with a 2D-CNN, which performs convolution over both the time and feature dimensions, did not yield consistent improvements. This may be due to the relatively low correlation between glottal parameters. Similarly, when the global average glottal features were used as input to a DNN, performance generally declined, indicating that short-term temporal variations in participants’ phonation may influence the discriminative capacity of 1D-CNN when processing frame-level glottal features.

#### 4.2.2 Fusion model performance testing

Based on the optimal parameter settings obtained from the aforementioned experiments, we conducted fusion experiments involving multiple modules. The experimental results are summarized in Table 7. The “w/o Fusion branch” configuration indicates that all frame-level feature elements are directly summed and used as the model output.

**Table 7:** Comparison of multi module fusion performance (%).

Module	Independent vowel features						Fusion branch			
	/a/	/o/	/e/	/i/	/u/	/ü/	w/o	w/ α	w/ β	w/ α+β
DNN	70.04	68.46	69.12	67.56	67.92	70.23	69.35	71.18	/	/
1D-CNN	72.65	73.86	70.92	74.13	72.65	73.93	74.24	76.12	/	/
2D-CNN	71.65	72.65	73.38	72.03	72.31	72.56	73.82	75.66	/	/
DNN+1D-CNN	72.75	73.86	70.43	74.26	73.17	72.92	74.36	76.90	76.82	78.76
DNN+2D-CNN	71.86	73.45	74.56	71.89	72.81	72.80	73.57	75.42	75.83	77.74
1D-CNN+2D-CNN	73.59	74.22	73.57	75.72	71.18	71.83	74.44	77.43	78.69	79.42
DNN+1D-CNN+2D-CNN	74.66	75.32	75.80	74.82	74.27	73.11	75.76	78.55	79.76	80.48

When evaluated independently, none of the single vowel features achieve consistently high accuracy across all models, indicating that vowel-specific discriminative capacity varies across different acoustic representations. For example, the DNN attains its best single-vowel performance with /a/ (70.04%), while the 1D-CNN performs best on /i/ (74.13%) and the 2D-CNN on /e/ (73.38%). This variation highlights the pronunciation-dependent discriminative capacity of different acoustic features.

For pairwise model combinations, simple feature concatenation (w/o fusion branch) provides only limited improvements. This observation suggests that direct feature concatenation fails to effectively exploit complementary information across different feature representations. By introducing the proposed fusion branches, consistent performance gains can be observed across all multi-model configurations. Specifically, incorporating fusion branch α improves the accuracy of DNN+1D-CNN from 74.36% to 76.90%, while 1D-CNN+2D-CNN increases from 74.44% to 77.43%. This improvement demonstrates that the fusion branch α is capable of capturing cross-feature interactions and selectively enhancing discriminative representations. Similarly, the introduction of fusion branch β can also improve the performance, as this branch models the complementary feature dependencies from an alternative perspective.

Finally, the dual-branch fusion strategy (α+β) achieves the best performance across all combinations. In particular, the triple-model configuration (DNN+1D-CNN+2D-CNN) reaches the highest accuracy of 80.48%, improving from 75.76% without the fusion branch. These results indicate that the two fusion branches provide complementary modeling capabilities, enabling the network to more effectively capture cross-feature correlations and vowel-dependent pathological cues. Furthermore, a closer inspection of the single-vowel contributions reveals that /a/ and /i/ consistently offer stronger discriminative information across multiple models, suggesting that these vowels are particularly sensitive to T2DM-related vocal changes.

To validate the effectiveness of the proposed method, we compare it with both traditional machine learning approaches and classical deep learning models. Specifically, K-Nearest Neighbors (KNN) (14) and Support Vector Machine (SVM) (15) are selected as representative traditional classifiers. For deep learning-based baselines, we employ Long Short-Term Memory (LSTM) (16) and VGG (17). For KNN and SVM, we use the handcrafted eGeMAPS feature set as input. For LSTM and VGG, two types of acoustic representations are evaluated, including MFCC (w/M) and Glottal (w/G). As shown in Table 8, deep learning-based methods consistently outperform traditional machine learning models. Among the deep learning baselines, LSTM and VGG demonstrate competitive performance under both MFCC and Glottal feature. Notably, the proposed method achieves the best overall performance. This improvement can be attributed to its ability to jointly integrate and model heterogeneous acoustic feature representations, thereby capturing more complementary information than single-feature-based baselines.

**Table 8:** Performance comparison (%) of different models.

Module	KNN	SVM	LSTM w/G	VGG w/G	LSTM w/M	VGG w/M	Ours
Accuracy	60.46	63.51	73.72	74.30	71.08	74.56	80.48

#### 4.2.3 Multidimensional information analysis of patients

Furthermore, we conducted a comprehensive evaluation of the predictive performance of the proposed framework across distinct demographic subgroups of T2DM patients, stratified specifically by gender and age. The results, summarized in Table 9, reveal important variations in classification efficacy linked to these demographic factors, where “Duration 0” represents non-diabetic group.

**Table 9:** Multidimensional information analysis of T2DM patients.

Parameters	Age subgroup											
	18–39				40–69				70 +			
Duration (years)	0	≤4	5–10	>10	0	≤4	5–10	>10	0	≤4	5–10	>10
Number (male/female)	13/9	2/3	0	1/0	65/65	22/22	27/19	4/7	30/35	7/12	9/10	10/6
Performance of male	76.92	50.00	/	0	80.00	86.36	81.48	75.00	90.00	85.71	88.88	100.00
Performance of female	88.89	66.67	/	/	73.84	77.27	73.68	71.42	80.00	83.33	90.00	83.33
Overall performance	82.91	58.34	/	0	76.92	81.82	77.58	73.21	85.00	84.52	89.44	91.67

Notably, across different age groups, the voices of male T2DM patients were generally more distinguishable than those of female patients, which contrasts with the findings reported in (18), possibly due to linguistic differences. Moreover, when considering the interaction between age groups and disease duration, longer duration of T2DM appeared to have no significant impact on vocal quality. It was observed that diabetes markedly affects vocal quality in older patients, and non-T2DM individuals in higher age groups also exhibited more distinguishable vocal features, which may be attributed to a synergistic effect between aging itself and diabetic pathology. Due to limited sample sizes in some demographic subgroups, subgroup performance should be interpreted cautiously and considered exploratory rather than confirmatory.

**Figure 6:**
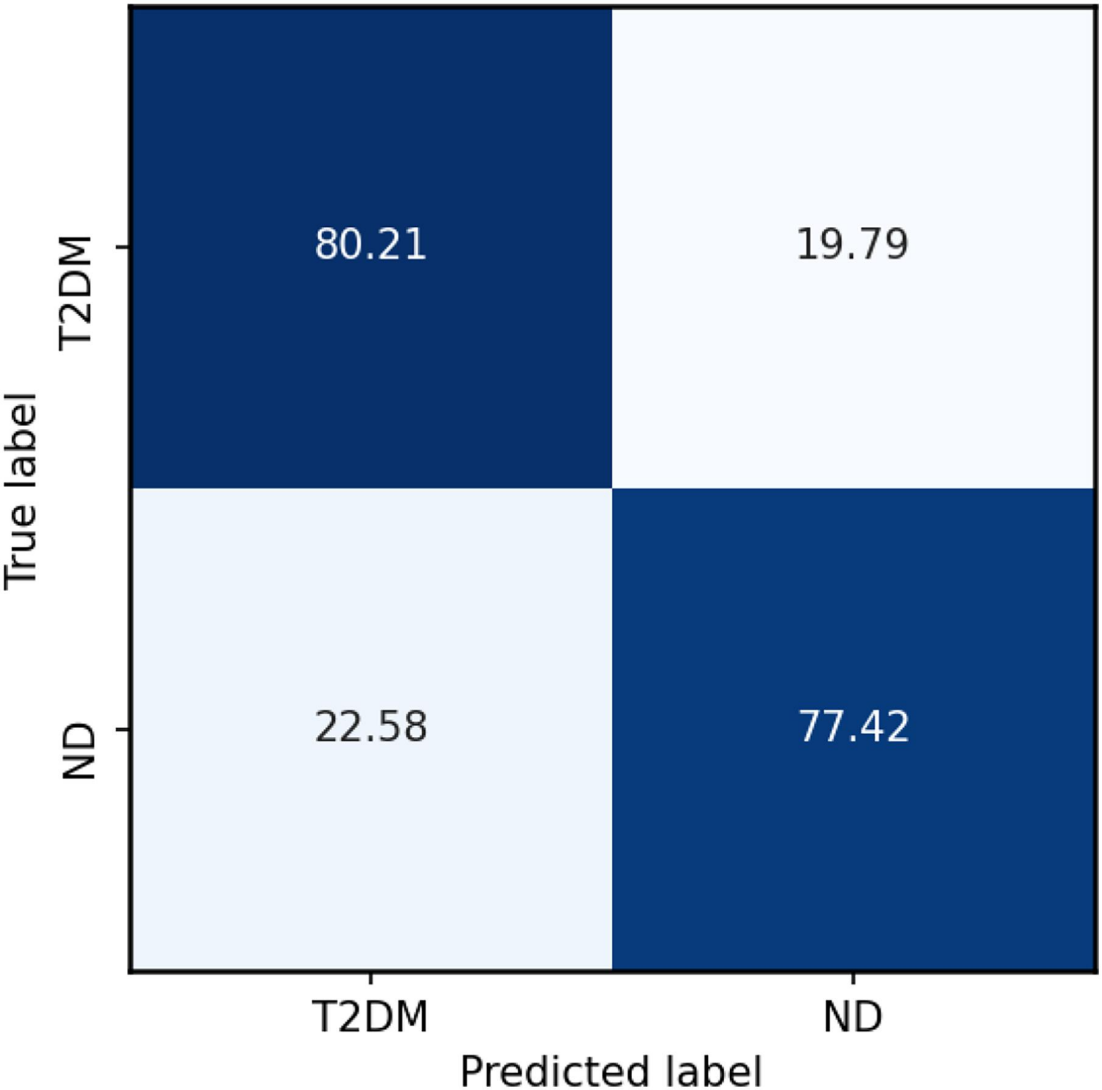
Confusion matrix (%) of the proposed model.

Additionally, class imbalance may have introduced bias into the analysis. For instance, in the 40–69 and 70+ age groups, the number of T2DM patients with a disease duration exceeding ten years was only 11 and 16, respectively, compared to 44 patients under 5 years in the 40–69 age group. Despite the limitations observed in certain disease-duration subgroups, these issues do not undermine the main diagnostic conclusions of this study. The proposed model was primarily evaluated using the overall cohort and age-stratified analyses with sufficient sample sizes, where consistent and robust performance was achieved across multiple evaluation metrics. In addition, the confusion matrix and the corresponding evaluation metrics are presented in Figure 6 and Table 10. The confusion matrix was generated from participant-level out-of-fold predictions across the five folds. Each participant contributed one prediction obtained from the fold in which they were assigned to the test set. These results demonstrate that the proposed method achieves a favorable balance between sensitivity and specificity. Because PPV and NPV depend strongly on disease prevalence, the values obtained from this balanced cohort should not be directly interpreted as population screening performance.

**Table 10:** Confusion matrix statistics and derived performance metrics.

Metric	Formula	Value
True Positives (TP)	–	154
False Negatives (FN)	–	38
False Positives (FP)	–	42
True Negatives (TN)	–	144
Sensitivity (Recall, TPR)	TP/(TP+FN)	0.802
Specificity (TNR)	TN/(TN+FP)	0.774
Area Under the ROC Curve (AUC)	/	0.794
Positive Predictive Value (PPV)	TP/(TP+FP)	0.791
Negative Predictive Value (NPV)	TN/(TN+FN)	0.785

Furthermore, this study has several limitations. First, the dataset exclusively comprises Mandarin vowel recordings, which may constrain the cross-lingual and population-level generalizability of the proposed T2DM detection model, particularly when applied to speakers of different languages and more diverse demographic cohorts. Therefore, further validation on multilingual and heterogeneous datasets is required to better evaluate the robustness of the proposed approach. Second, although the hierarchical network with feature-level fusion demonstrates promising performance, its applicability still needs to be validated on larger-scale datasets to fully assess model effectiveness. The current model lacks detailed interpretability analysis. Future work will incorporate explainable AI methods, such as attention visualization, SHAP analysis, and feature attribution methods, to identify acoustic patterns contributing to classification.

## 5 Discussion

This study developed a Mandarin voice-based framework for T2DM-related classification. By integrating multiple acoustic representations, the proposed model achieved an overall classification accuracy of 80.48%, demonstrating the feasibility of voice-based analysis as a potential auxiliary approach for T2DM-related screening.

Previous studies investigating the relationship between voice characteristics and T2DM have mainly focused on identifying acoustic differences and exploring potential disease-related voice biomarkers. In this study, we further extended previous findings by demonstrating that multiple acoustic characteristics extracted from Mandarin vowel phonations can be effectively integrated for T2DM classification, providing additional evidence supporting the feasibility of voice-based approaches for T2DM screening. Consistent with previous studies (7, 8), our results confirmed significant differences in acoustic features between individuals with T2DM and non-diabetic controls, suggesting that voice signals may contain potential markers associated with T2DM. Similar to the findings reported in (19), our analysis indicated that older T2DM participants were more likely to be correctly identified by the proposed model. This observation may suggest that age-related physiological changes could amplify diabetes-associated vocal alterations. However, this finding should be interpreted cautiously, as the limited number of older participants in our dataset may also influence the observed performance differences. Previous research (4, 18) has reported that female individuals with T2DM may be more easily detected using voice-based approaches. In contrast, our results did not demonstrate the same tendency. This discrepancy may be related to differences in language characteristics, speech tasks, recording protocols, and population distributions.

Although our study provides preliminary evidence for automated T2DM detection using voice signals, current research in this area remains at an early stage. Future studies should focus on expanding existing datasets, conducting multi-centre validation, investigating the biological mechanisms underlying T2DM-related voice changes, and developing more robust automated models that can be evaluated under real-world conditions.

## Data Availability

The raw data supporting the conclusions of this article will be made available by the authors, without undue reservation.
